# The short-run causal effect of tumor detection and treatment on psychosocial well-being, work, and income

**DOI:** 10.1007/s10198-015-0688-7

**Published:** 2015-04-05

**Authors:** Sofie J. Cabus, Wim Groot, Henriëtte Maassen van den Brink

**Affiliations:** Top Institute for Evidence Based Education Research, TIER-Maastricht University, Kapoenstraat 2, 6211 KW Maastricht, The Netherlands; Amsterdam School of Economics, University of Amsterdam, Roeterstraat 11, 1017 LW Amsterdam, The Netherlands

**Keywords:** Income, Psychosocial well-being, Tumor detection, Treatment, Work, I2

## Abstract

This paper estimates the short-run causal effect of tumor detection and treatment on psychosocial well-being, work and income. Tumor detection can be considered as a random event, so that we can compare individuals’ average outcomes in the year of diagnosis with the year before. We argue for using panel data estimation techniques that enable us to control for observed and unobserved information intrinsic to the individual and time constants. We use data of a national representative panel in the Netherlands that includes health survey information and data on work, education, and income between 2007 and 2012. Our findings show differences in the psychosocial dysfunction of men and women in response to tumor detection and treatment. Women, not men, are decreasingly likely to participate in the labor force as a result of malignant tumor detection, while no significant effects are found on her personal or household income. We also demonstrate that fixed effects panel data models are superior to matching techniques.

## Introduction

The Health and Consumer Protection Directorate-General [12] have argued in their guidelines for “Quality Assurance in Breast Cancer Screening and Diagnosis” that almost 10 in every 100 individuals (9 %^1^) from 1985 to 2000 can be saved thanks to screening policies particularly targeting specific sites: breast, cervical and colorectal cancers.^2^ However, we do not fully understand how tumor detection through screening and treatment impacts patients’ daily lives. A review of the literature points out that authors mainly focus on coping strategies of patients who have (recently) been diagnosed with cancer. They do not, or only to a limited extent, consider direct measures of subjective psychosocial well-being (see Sect. “Background”). Moreover, previous studies dealing with coping strategies or the financial implications of tumor detection have various methodological shortcomings [30]. These methodological shortcomings include (Cook and Campbell [11]): sample selection issues; limited sample size; descriptive research or estimating correlations; omitted variables bias; and external validity. Research into tumor detection and treatment and its effect on individual outcomes often relies on administrative or survey data from hospitals or clinics. It also relies on matching estimation techniques that can identify an appropriate counterfactual outcome for every individual case based on a set of observable characteristics.

This paper contributes to the previous literature in at least three ways. First, we use a national representative panel with information both after and before tumor detection. In particular, studies using the follow-up of various health and labor-related outcomes before tumor detection are, to our best knowledge, rare in the economics literature, as one cannot anticipate tumor detection. Only two papers, Moran et al. [21] and Heinesen and Kolodziejczyk [16], use an alternative control group made up of currently healthy individuals who developed cancer 5 years later. But this paper benefits from data from a national representative panel. It consists of individuals who were selected from the population register by Statistics Netherlands, and combines rich health survey data with administrative data on work and income on a year-to-year basis [28]. We argue for using panel data estimation techniques instead of matching or correlational analysis, and will use both techniques in this paper in order to support our arguments. Panel data techniques allow us to construct a ‘control group’ from the data by using individuals in the year before diagnosis who have by construction no prior knowledge of their disease status in the upcoming year. Second, we include a varied set of measures of subjective well-being available in the panel, namely: self-reported health, self-assessed happiness, anxiety, depression, and hindrance at work. With respect to financial well-being, we look at the ‘traditional’ variables like labor force participation and (log of) wages. And third, the data allow us to distinguish similar outcomes by gender. Previous literature indicated that, owing to the small sample sizes, splitting up the results by gender was difficult (e.g. [21]).

## Background

### Subjective psychosocial well-being

We searched for articles meeting a set of inclusion criteria, explained below, that do not necessarily cover the whole range of the literature on the relationship between cancer and psychosocial well-being, as this was considered beyond the scope of this article. It was of primary importance that we position our problem statement of the effects of tumor detection on subjective psychosocial well-being in the literature, as well as to search for articles using a systematic approach. As such, we started our literature review with the following keywords in PubMed and Google Scholar: “anxiety”; “cancer”; “diagnosis”; “happiness”; “hospitalization”; “life satisfaction”; “psychosocial”; “well-being”; “quality of life”; and “tumor” or “tumour”. We also searched for articles in, for instance, Science Direct. However, most of these articles deal with the effects of medical or treatment implications of being diagnosed and treated for cancer instead of measures of subjective well-being. Therefore, we decided to start mainly from the PubMed and Google Scholar search results. The second inclusion criterion for the literature search is publication between the years 2000–2014. Third, we only retained articles that had been published in peer-reviewed journals, and that were written in the English or Dutch language. Furthermore, the articles should especially deal with cancer or tumor detection, and its impact on subjective psychosocial well-being, preferably in the short-run. Doing so, we retained only seven articles that met all four inclusion criteria (Table 1). Of these 7 articles, 6 dealt with breast cancer (BrC).

**Table 1:** Tumor detection and treatment and its relationship with psychosocial well-being

References	Country	*N*	Type cancer	Data collection	Main findings
Sammarco [27]	US	101	BrC	Survey	Role of social support in QoL
Wonghongkul et al. [31]	Thailand	150	BrC	Survey at one hospital	Coping by seeking social support
Arora et al. [3]	US	246	BrC	Survey at two hospitals	Information and social support decrease over time
Kotkamp-Mothes et al. [20]			Lump together	Literature	Sustain autonomy of elderly by including relatives in social support programs
Aukst-Margetić et al. [2]	Croatia	115	BrC	Survey at one hospital	Role of religiosity in depression
Reddick et al. [23]	US	138	BrC	Clinical trial	Role of coping strategies in depression, anxiety and fatigue
Raque-Bogdan et al. [22]	US	13	BrC	Interviews	BrC intensified need for purpose in life/work

*BrC* breast cancer

Most studies focus on “coping strategies”, for example how cancer patients cope with their illness in daily life. A survey study at one hospital in Thailand presents several coping strategies [31]. Among these strategies, seeking social support had been indicated the most. Sammarco [27] discusses the positive correlation between (perceived) social support and quality of life of younger breast cancer (BrC) patients in the US. Other evidence from the US (Chicago) from patient surveys indicates that social support is highest close to tumor diagnosis, but then it significantly decreases over time [3]. Kotkamp-Mothes et al. [20] add that, in some cases, patients refuse social support, particularly the elderly, if it is an impediment to their autonomy.

There is one recent qualitative study from Raque-Bogdan [22] that deals with the coping of young BrC survivors. She indicates that this group significantly differs from the elderly, as they are in need of reintegration into the labor force after treatment. From the interviews, the author synthesized that these young women increasingly seek purpose in life, sometimes in their private life, but most often in their work. However, Raque-Bogdan [22] also indicates that this is not always possible due to difficulties with changes in work and financial insecurity. Another study [2] confirms this purpose-seeking behavior as a coping strategy, and explores the role of religion. The authors point to the meaningfulness of religion in reducing the risk of depression. Reddick et al. [23] conducted a clinical trial in the US, also to explore the role of several coping strategies in depression as well as anxiety and fatigue. They also argue that ‘better’ coping strategies go hand-in-hand with increased psychosocial well-being.

Based on Cook and Campbell [11], we indicate that the evidence and the methodological quality of the traced research studies in Table 1 are rather poor: the sample size is low, and most studies violate external validity by reporting on data from one hospital (e.g. [31]) or two hospitals (e.g. [3]). Making inferences about the internal validity is difficult, as the surveys used in the studies are not available. Furthermore, there is only one randomized, controlled, clinical trial [23].

### Work and income

Starting from the literature review of Steiner et al. [30], we undertook a different review strategy than described in subsection “Subjective psychosocial well-being”. In fact, we only added four articles that were more recently published in peer-reviewed journals to the review of Steiner and co-authors. These articles have been collected following the same approach and inclusion criteria as previously discussed.

Before turning to the results summarized in Table 2 we first describe an earlier US study of Bradley et al. [6]. This study focuses on breast cancer as a single disease as well as its impact on employment decisions and earnings up to 3 years after the initial diagnosis. The authors find that women who survive up to 3 years after initial diagnosis, are less likely to be in the labor force (about 50 % compared to over 60 %). However, those who are still in the labor force work more and earn higher wages than their peers without breast cancer. They discuss that the way health insurance coverage is provided in the US largely drives their results. Moran et al. [21] point to the relatively old ages (mean age of 56) of individuals in the sample used in Bradley et al. [6]. They add to the US literature by focusing on the effects of surviving cancer on the labor market outcomes of individuals aged 28–54. Their case group is compared with a control group constructed from the Panel Study of Income Dynamics. Moran et al. [21] indicate an overall reduction in the employment rate and hours of work of comparable magnitude to those estimates found for older workers in Short et al. [29]. This overall reduction in the employment rate of cancer survivors may be owing to early retirement (e.g. [9]). Moran et al. [21] additionally find that, in the long-run, individuals who relapse have worse labor market outcomes. Workers with relapses may benefit more from employment support services and workplace accommodation, as indicated by Short et al. [29].

**Table 2:** Tumor detection and treatment and its relationship with work and income

References	Country	Sample	Type cancer	Data collection	Main findings
Carlsen et al. [9]	Denmark	44,905	Selection	Population based cohort study	Risk of early retirement
Heinesen and Kolodziejczyk [16]	Denmark	7371	Selection	Cancer Registry	Risk of unemployment and disability pension
Moran et al. [21]	US	1800	Lump together	Longitudinal survey at one hospital, combined with PSID survey	Lower employment rates and work fewer hours
Short et al. [29]	US	504	Lump together	Hospital combined with HRS survey	Persons with recurrences or second primary tumors may particularly benefit from employment support services and workplace accommodation

*HRS* Health and Retirement Study, *PSID* panel study of income dynamics

In contrast to the literature review of subsection “Subjective psychosocial well-being”, Table 2 presents studies with better methodological quality, especially when the authors combine hospital data with administrative and/or survey data (e.g. [29]). However, several issues remain unresolved. First, respondents self-select themselves into the data, as they are only included in case of illness and treatment at the hospital (e.g. [24]). As a result of this data collection method, there is no information available about past events without the respondents having prior knowledge about their health status. Information obtained at the time of the first medical consultation may be severely biased by having this knowledge. A second issue arising in most case–control studies is the absence of a good control group. Often, researchers rely on matching techniques that can identify an appropriate counterfactual outcome for every individual case based on a set of observable characteristics (e.g. [9, 21]). The set of control variables often only captures information on limited background characteristics (e.g. Heinesen and Kolodziejczyk [16]). But even with good control variables, matching analysis does not appropriately deal with the influence of genetic susceptibility, particularly in a study on why individuals develop cancer. As such, finding a good control group is almost an impossible mission (see also [30]).

## Empirical strategy

The best control group one can think of is the case individual his- or herself, but in a “healthy” state. In fact, tumor detection can be considered a sudden shock, which seriously can affect the individual’s well-being in the year of detection, especially compared with the year before [6, 7]. Two important assumptions underlie this empirical strategy. First, observed and unobserved background characteristics may not determine the level and change of well-being (i.e. endogeneity). We deal with this econometric challenge by estimating an individual and time fixed effects model. This estimation model controls for information intrinsic to the individual and time constants. And second, individuals may not anticipate tumor detection in the year before the actual diagnosis is made. Bradley et al. [6, 7] already argued that tumor detection can be treated as a random event. We check for this assumption in Sect. “Robustness of the results”.

### Modeling well-being

We model the well-being function $W_{\text{it}}^{*}$ as (Groot and van den Brink [14]):

1 $$W_{\text{it}}^{*} \sim \left( {{\text{HS}}_{\text{it}} ;\;Y_{{i\left( {t - 1} \right)}} ;\;X_{\text{jit}} } \right),$$

where *W*_it_ denotes individual *i*’s well-being at time *t*; *HS*_it_ the health status; *Y*_*i*(*t*−1)_ income in the year before diagnosis; and *X*_jit_ a vector of $j \in \{ 1,2, \ldots ,J\}$ background characteristics at time *t*.

In the case of tumor detection, HS_it_ depends on:

2 $${\text{HS}}_{\text{it}} \sim \left( {D_{\text{it}} ;N_{\text{it}} ;H_{\text{it}} } \right),$$

where *D*_*it*_ denotes detection with 1 ‘tumor detected’, and 0 otherwise; $N_{\text{it}} \in \{ 0,\;1\}$ the nature of the tumor with 1 ‘benign’, and 0 ‘malignant’; and *H*_it_ information on hospitalization.

As in Groot and Maassen van den Brink [14], it is assumed that well-being is a linear function of lagged income (*Y*_it−1_), health status (*D*_it_; *N*_it_; *H*_it_), and other background characteristics (*X*_jit_). We then may estimate:

3 $$W_{it}^{*} = \gamma_{0} + \gamma_{1} \left( {D\left( 1 \right)_{it}^{*} \times H\left( 0 \right)_{\text{it}} } \right) + \gamma_{2} \left( {D\left( 0 \right)_{\text{it}} \times H\left( 1 \right)_{\text{it}} } \right) + \gamma_{3} \left( {D\left( 1 \right)_{\text{it}} \times H\left( 1 \right)_{\text{it}} } \right) + \gamma_{4} N_{\text{it}} + \gamma_{5} \;{\text{Age}}_{\text{it}} + \gamma^{6} Y_{{{\text{it}} - 1}} + \mathop \sum \nolimits \delta_{j} X_{\text{jit}} + \varepsilon_{\text{it}} ,$$

by using individual fixed effects models.

Depending on the nature and stage of the tumor, we expect large heterogeneity of the treatment effect. For instance, tumors that are considered benign do not always have to be removed, and tumors that are diagnosed as malignant do not always imply severe medical therapy. Therefore, three interaction terms are included in Eq. (3): (1) tumor detection without hospitalization $D\left( 1 \right) \times H(0)$; (2) only hospitalization $D\left( 0 \right) \times H\left( 1 \right)$; and (3) tumor detection including hospitalization $D\left( 1 \right) \times H\left( 1 \right)$. Hence, the reference category is $D\left( 0 \right) \times H\left( 0 \right)$, i.e. no tumor detection and no hospitalization.^3^ Age is also included in Eq. (3), so as to control for the negative relationship between age(ing) and well-being. Note that, by estimating Eq. (3), time invariant *X*_jit_ is dropped because of obvious reasons of multicollinearity.

Groot and Maassen van den Brink [13] argue that measures of self-reported life satisfaction (e.g. happiness) are affected by preference drift. Or else, in case individuals experience a life-changing event, such as tumor detection, they likely mirror their personal/income situation with those of others in the same situation. We account for preference drift in two ways. First, our results with respect to psychosocial well-being are controlled for income in the year before diagnosis *Y*_it−*1*_, in particular, we cluster individuals by using dummies of the lagged values of the net personal income categories. Second, both models on well-being, work and income include the variable ‘nature of the tumor’.

### Modeling earnings

We model the earnings function $Y_{\rm {it}}^{*}$ as [6]:

$$Y_{\text{it}}^{*} \sim \left( {{\text{HS}}_{\text{it}} ;S_{\text{it}} ;{\text{HI}}_{\text{it}} ;X_{\text{jit}} } \right),$$

where *S*_it_ denotes the duration of surviving a tumor diagnosis to date; HI_it_ information on health insurance; and *X*_jit_ individual, family, neighborhood characteristics and life-style factors. As we focus on the short-run, *S*_it_ is equal to 1 year for all individuals.

To estimate the earnings function, we replace $W_{\text{it}}^{*}$ by $Y_{\text{it}}^{*}$, and include age as a continuous variable. Bradley et al. [6] suggest additionally controlling for an age dummy, instead of its squared term, so as to account for the age at which individuals retire. We include an age dummy indicating 65+, as this was the retirement age in the Netherlands before 2013.

Contrary to Bradley et al. [6], we do not include information on health insurance into the convenient regression. Inhabitants of the Netherlands can change their coverage only once a year on January 1. Consequently, HI_it_ should drop out of estimation owing to multicollinearity. If not, this would directly falsify our assumption of no anticipation (Sect. “Robustness of the results”).

## Data

We use data of the LISS (Longitudinal Internet Studies for the Social Sciences) panel administered by CentERdata (Tilburg University, the Netherlands). The LISS panel is a representative sample of Dutch individuals who participate in monthly Internet surveys. The panel is based on a true probability sample of households drawn from the population register. Households that could not otherwise participate are provided with a computer and internet connection. A longitudinal survey is fielded in the panel every year, covering a large variety of domains including work, education, income, housing, time use, political views, values and personality. More information about the LISS panel can be found at: http://www.lissdata.nl [28].

The LISS data on health include 7000–9000 households each year. There have been six waves in total over the period 2007–2012. In the first wave of the year 2007, 8478 households (100 %) were asked to fill in the questionnaires on health and well-being. CentER data and Statistics Netherlands select households to participate in the study. As such, households cannot self-select themselves into the survey. The response rate was 78.9 % (6625 households) in 2007. Overall, there was a high response rate in 2008 (72.0 % of 8280 households), in 2009 (66.7 % of 9170 households), in 2010 (77.6 % of 7364 households), in 2011 (77.6 % of 6533 households), and in 2012 (85.4 % of 6769 households). As such, the data include 44,741 observations. After elementary data cleaning of the variables further used and discussed in this paper, we still kept 33,870 observations in the sample. Most of these observations are the head of the household (53.9 %), wedded partner (29.1 %), or unwedded partner (5.5 %). There is some information available about a child living at home (10.6 %), or any other kind of family member or housemate (about 1.0 %).

Each year in the month of November, respondents were asked to answer the following question: “Has a physician told you this last year that you suffer from: (1) cancer or malignant tumor, including leukemia or lymphoma, but excluding less serious forms of skin cancer; and/or (2) skin tumor, polyps, angioma”. Those respondents, who answered “yes”, constitute the case group, a total sample of (*N* = 1102) observations. Table 3 presents the total number of individuals from which we know the disease status in year t and in year *t* − 1. Whereas the mean observed time for those respondents with a tumor diagnosis is 2.7 years (SD 1.5, minimum 1 and maximum 6), we only lose respondents who are only observed once in the data, in total 252 observations. The final sample of case–control individuals then consists of (*N* = 850) observations, of which exactly 425 are unique individuals before and after tumor detection (see Table 3).^4^ Tables 10 and 11 in the Appendix summarize the rich data we have on background characteristics.

**Table 3:** Control group—follow up

	2007	2008	2009	2010	2011	2012	Total
Control group—follow up							
*D*(0)	92	87	75	83	88	0	425
*D*(1)	0	92	87	75	83	88	425
Total	92	179	162	158	171	88	850
Control group—before matching							
*D*(0)	6609	5791	5964	5572	4931	5692	34,559
*D*(1)	0	92	87	75	83	88	425
Total	6609	5883	6051	5647	5014	5780	34,984
Control group—after matching							
*D*(0)	46	59	52	73	94	95	419
*D*(1)	0	92	84	74	81	88	419
Total	46	151	136	147	175	183	838

In our data, there are about 85 individuals each year who are diagnosed with a tumor, with 54 % having a benign tumor (and therefore 45 % having a malignant tumor). This corresponds to an average cancer incidence rate of about 677 per 100,000 individuals each year 2008–2012. For reasons of comparability, the official Dutch statistics of the cancer registry (cijfersoverkanker.nl) are presented in Table 4. The cancer registry indicates an average cancer incidence rate of 584 per 100,000 individuals each year over the same period. These numbers are highly comparable with those of LISS. The Kolmogorov–Smirnov equality-of-distributions test looks at the distribution of cancer incidence observed in the LISS sample and compares it with the distribution of cancer incidence from the official Dutch statistics. The results from this test confirm the comparability of both samples (*D* = 0.6000; *P* value = 0.329).

**Table 4:** Comparability of Statistics Netherlands and Cancer Registry and the LISS panel data

Population Netherlands^a^				LISS panel			
Year	Total population	# Cancer incidence	# Per 100,000	Full sample	# Tumor incidence^b^	Cancer? (yes = 1)	# Per 100,000
2008	16,485,787	91,688	556	5962	92	30	503
2009	16,574,989	93,971	567	6116	87	40	654
2010	16,655,799	97,412	585	5714	75	30	525
2011	16,730,348	101,833	609	5070	83	45	888
2012	16,779,575	101,210	603	5781	88	47	813
# Per 100,000			584				677

^a^Own handling of Statistics Netherlands (CBS.nl) and Cancer Registry (cijfersoverkanker.nl)

^b^Tumor incidence denotes malignant as well as benign tumors

Table 5 summarizes the outcome variables including comparability statistics. The descriptive statistics of the control variables including the individual background characteristics of the respondents and several determinants of life-style are available in the “Appendix”.

**Table 5:** Outcomes, treatment variables and comparability statistics (full sample, *N* = 850)

	Disease status		Difference	*T* value
	*D*(0)	*D*(1)	*D*(0) − *D*(1)	
Individual outcomes				
Self-reported health (5-point Likert scale)	2.9	2.6	0.3	4.79
Happiness (5-point Likert scale)	4.2	4.0	0.2	2.15
Anxiety (5-point Likert scale)	2.2	2.5	−0.3	−3.19
Hindrance (at) work (5-point Likert scale)	2.0	2.4	−0.4	−4.26
Income and work				
Unable to go to work (days)^a^				
0 days	0.7316	0.5953	0.1363	3.97
1 or 2 days	0.1032	0.1059	−0.0026	−0.12
3–5 days	0.0796	0.0871	−0.0074	−0.37
5–10 days	0.0295	0.0588	−0.0293	−1.93
More than 10 days	0.0560	0.1529	−0.0969	−4.30
Has no paid labor (yes = 1)	0.5597	0.5412	0.0185	0.48
Household net monthly income (log)	7.7198	7.7270	−0.0071	−0.19
Tumor detection				
Benign (yes = 1)	0.0000	0.5482	−0.5482	−19.83
Hospitalization				
Hospitalization (%)	0.12	0.4306	−0.3106	−10.8
Days in the hospital (per year)	0.6	2.5	−1.9	−6.22

^a^the total number of days that respondents were unable to go to work, perform the housekeeping or study

This paper uses direct measures of subjective psychosocial well-being, such as: self-reported health, happiness, anxiety, and hindrance at work. The choice for this set of variables in order to measure subjective well-being is based on a long-standing tradition of (health) economists (for an elaborated discussion on this, see Kahnenman and Krueger [17]). Briefly, it is argued that direct measures of subjective well-being, such as self-reported happiness, are particularly useful in the measurement of social welfare. For example, Gruber and Mullainathan [15] use self-reported happiness as an outcome measure in their study on the effects of cigarette taxes on subjective psychosocial well-being. Kling et al. [19] evaluated the effects of a program that offered housing vouchers to high-poverty families living in the US on self-reported physical and mental health. These health states were measured by survey questions directly asking about feelings of being calm and peaceful, anxiety, and depression. Kahnenman and Krueger ([17], p. 4) further discuss that individuals’ perceptions of their experiences are most accurately measured when reported close to the actual experience. As the survey asks the respondent exactly the same question in two subsequent years in the month of November, we argue that our measures of well-being will reflect the way people feel about experiences that are still fresh in their minds (see also [6]). Further, note that we prefer direct measures of subjective well-being above composite measures such as quality of life, or life satisfaction, for two reasons. First, measures like quality of life are often composed from many different underlying questions aiming at grasping the full concept. These questions often differ from survey to survey, and, therefore, the measure is subject to problems with internal and often also external validity and reliability. Second, quality of life and life satisfaction can be approximated by direct measures (e.g. like self-reported happiness or hindrance at work) that do not suffer, or only in a limited way, from these issues (Cook and Campbell [11]). Even though reliability of direct measures can be subject to context, mood, and duration neglect, “the idiosyncratic effects of recent, irrelevant events are likely to average out in representative population samples (Kahnenman and Krueger [17], p. 7)”.

First, we discuss the variable self-reported health that has been derived from the question: “How would you describe your health, generally speaking?” Respondents could give their answer on a five-point Likert scale ranging from poor (answer = 1) to excellent (answer = 5). We observe a significant overall decline in self-reported health status before and after diagnosis.

Next, the respondents were asked about their feelings and emotions over the past month by answering the question: “The following questions are about how you felt over the past month. For every question, please choose the answer that best describes how you felt during this past month. This past month…”. This question was asked for: happiness “I felt very happy“, and anxiety “I felt very anxious”. Respondents could answer on a five-point Likert scale ranging from never (answer = 1) to continuously (answer = 6). From Table 5, we observe an overall decline in happiness and an overall increase in anxiety in the year of tumor detection.

Third, we also consider a question related to work: “To what extent did your physical health or emotional problems hinder your work over the past month, for instance in your job, the housekeeping, or in school?”. Respondents could answer on a five-point Likert scale ranging from not at all (answer = 1) to very much (answer = 5). Individuals increasingly find that their physical health or emotional problems hinder work, the housekeeping or school, illustrated with a change from 0 to 1 in disease status.

Fourth, we provide information on personal net monthly income (in logarithm) and household net monthly income (in logarithm) for the full sample. We do not observe any significant difference before and after tumor detection.

The variables with respect to nature of the tumor ‘benign’, and hospitalization ‘hospital’, are also summarized in Table 5. First, out of all of the tumors diagnosed, roughly half are benign. Of course, in the control group no (0.00 %) tumors are diagnosed. Second, respondents were asked the following question: “Did you spend any time in hospital or a clinic over the past 12 months?” In total 12.0 % (*N* = 51) of the respondents said “yes” if (*D* = 0), and 43.1 % (*N* = 183) if (*D* = 1). The respondents were also asked how many days they had to stay in the hospital throughout the year. The control group reports less than 1 day, while the case group reports 2.5 days. In addition to this information, respondents could indicate whether or not they were hospitalized for an operation over the past year. In the control group, only 6.6 % said “yes”, while it was 33.4 % in the case group.

In order to check for overlap between the variables selected for the analyses, we have estimated a Pearson correlation matrix (available from the authors upon request). As expected, we observe a significant correlation between the variables. Highest correlation is found between the variables measuring self-reported health and hindrance at work and unable to work and hindrance at work. However, all of the correlations range from low to medium so that we conclude that each of the chosen variables still has a lot of variance unexplained, and, as such, add to the analyses.

## Main results

Table 6 summarizes the results of four models by gender with respect to: self-reported health (model 1); happiness (model 2); anxiety (model 3); and the extent to which the individual’s health problems hinder work, housekeeping or study (model 4). All of the outcome variables have been standardized, so that the results can be expressed as effect sizes (ES). The individual and time fixed models have been estimated using the ordinary least squares estimator. Robust standard errors are reported between brackets.

**Table 6:** Impact of tumor detection and treatment on subjective psychosocial well-being (estimation output of the fixed effects model)

	Model: health		Model: happiness		Model: anxiety		Model: hindrance (at) work	
	Women	Men	Women	Men	Women	Men	Women	Men
Reference *D*(0) × *H*(0)								
*D*(0) × *H*(1)	0.2625 (0.3341)	−0.1130 (0.2211)	0.2047 (0.3935)	0.0314 (0.2413)	0.1624 (0.4493)	0.8478 (0.2886)***	0.2619 (0.4022)	0.2240 (0.2965)
*D*(1) × *H*(0)	−0.7862 (0.2034)***	−0.5650 (0.2347)**	0.2175 (0.2396)	−0.1846 (0.2562)	0.1240 (0.2735)	0.4627 (0.3064)	0.3977 (0.2448)	0.2456 (0.3147)
*D*(1) × *H*(1)	−0.7442 (0.1776)***	−0.6727 (0.2296)***	−0.0482 (0.2091)	−0.4524 (0.2506)*	0.2368 (0.2387)	0.7967 (0.2997)***	1.0251 (0.2137)***	0.6266 (0.3079)**
Control variables	Age, benign, L. income	Age, benign, L. income	Age, benign, L. income	Age, benign, L. income	Age, benign, L. income	Age, benign, L. income	Age, benign, L. income	Age, benign, L. income
Obs.	341	320	341	320	341	320	341	320
Groups	210	199	210	199	210	199	210	199

Robust standard errors between brackets

Asterisk levels denote 1 % significance (***), 5 % significance (**) and 10 % significance (*)

First, consider the interaction effect between *D*(0) × *H*(1). Note that *D*(0) × *H*(0) is the reference category. Across the models, we do not find any significant effects of cancer treatment on self-reported health, happiness, and hindrance at work. The results, illustrated in model 3, indicate that, compared to the control group, hospitalization significantly increases anxiety among men with (ES = +0.8478).

Next, consider the interaction effect between *D*(1) × *H*(0). Tumor detection significantly decreases self-reported health among women (ES = −0.7862) and among men (ES = −0.5650). Consequently, tumor diagnosis has a moderate to large impact on self-reported health status. Note that, not controlling for the nature of the tumor, ‘benignʼ, we find a small positive effect size of +0.2826 in the model on happiness. As such, women who have been diagnosed with a benign tumor are considerably happier in the year of diagnosis compared to the control group (for a discussion, see also [1].

Third, consider the interaction effect between tumor detection and hospitalization [*D*(1) × *H*(1)]. Overall, for men, we observe a moderate to large impact of tumor detection and treatment on health (ES = −0.6727), happiness (ES = −0.4524), anxiety (ES = +0.7967), and hindrance at work (ES = +0.6266). For women, we only observe a significant effect on health status (ES = −0.7442) and hindrance at work (ES = 1.0251).

We conclude that, particularly among men, tumor detection and treatment have a strong and significant negative impact on emotions and feelings of happiness and anxiety. However, compared with the control group, the impact of tumor detection on hindrance at work is higher for women than for men.

Table 7 summarizes the results for work and income by gender. Four models have been estimated, namely: at least 1 day per month unable to go to work, perform housekeeping or study (1 = at least 1 day) (model 1); labor status (has no paid labor = 1) (model 2); log of net personal income (model 3); and log of net monthly household income (model 4).

**Table 7:** Impact of tumor detection and treatment on work and income (estimation output of the fixed effects model)

	Model: unable to work		Model: has no paid labor		Model: personal income		Model: household income	
	Women	Men	Women	Men	Women	Men	Women	Men
Reference *D*(0) × *H*(0)								
*D*(0) × *H*(1)	−0.1832 (0.1337)	−0.1716 (0.1028)*	0.0106 (0.0451)	0.0300 (0.0444)	0.0112 (0.0583)	0.0260 (0.0389)	−0.0340 (0.0514)	0.0284 (0.0470)
*D*(1) × *H*(0)	−0.2428 (0.1008)**	−0.0255 (0.0950)	0.1212 (0.0286)***	0.0220 (0.0419)	−0.0377 (0.0358)	0.0152 (0.0374)	0.0275 (0.0325)	0.0120 (0.0430)
*D*(1) × *H*(1)	−0.4190* (0.0881)**	−0.1569 (0.0919)*	0.1061 (0.0254)***	−0.0446 (0.0390)	−0.0064 (0.0332)	0.0075 (0.0346)	0.0077 (0.0300)	0.0260 (0.0402)
Control variables	Age, age sq., benign, d65+	Age, age sq., benign, d65+	Age, age sq., benign, d65+	Age, age sq., benign, d65+	Age, age sq., benign, d65+	Age, age sq., benign, d65+	Age, age sq., benign, d65+	Age, age sq., benign, d65+
Obs.	389	361	349	329	306	312	325	318
Groups	210	199	210	199	186	189	198	192

Robust standard errors between brackets

Asterisk levels denote 1 % significance (***), 5 % significance (**) and 10 % significance (*)

Again, we first consider the interaction effect *D*(0) × *H*(1), and observe that, for women, hospitalization does not significantly impact labor force participation or income. However, it does impact the ability to work for men (−17.16 % points).

Next, the effect of tumor detection without hospitalization is considered [*D*(1) × *H*(0)]. Compared with the control group, the results indicate that women are less able to work (−24.28 % points), or are increasingly likely to drop out of paid labor (+12.12 % points). The models do not report significant estimates for men.

For women, the same picture is sketched when hospitalization follows tumor detection [*D*(1) × *H*(1)], namely: women are less able to work (−41.90 % points) and are increasingly likely to drop out of the labor force (+10.61 % points). The results also indicate a significant negative impact on menʼs ability to work (−15.69 % points).

To conclude, overall, no significant impact of tumor detection and treatment [i.e. *D*(1) × *H*(1)] is found on personal or household income. Only for women without a partner, we estimated a significant decrease in household income by about −17.33 %.

## Robustness of the results

### Can matching analysis work?

The case individuals used for matching analysis are the same individuals as those of Table 3. Each year between the years 2007–2012, we have a large set of control individuals. Case individuals are then matched to “healthy” individuals based on a rich set of background characteristics *X*_jit_ (Tables 10 and 11 in Appendix) and by year. We deal with propensity score matching by using the convenient nearest neighbor matching (NNM) estimator without replacement and with random sorting of the data. The probit model is used for obtaining the propensity scores. After applying the propensity score matching, the ordinary least squares estimator is again used on the matched sample for model estimation. The standard errors are clustered at the individual level. The results of the NNM estimation models are summarized in Tables 8 and 9.

**Table 8:** Impact of tumor detection and treatment on subjective psychosocial well-being (estimation output of the nearest neighbor matching model)

	Model: health		Model: happiness		Model: anxiety		Model: hindrance (at) work	
	Women	Men	Women	Men	Women	Men	Women	Men
Reference *D*(0) × *H*(0)								
* D*(0) × *H*(1)	−0.2710 (0.2652)	−0.5071 (0.2230)**	−0.1822 (0.2588)	−0.2572 (0.2256)	0.0537 (0.2602)	0.0537 (0.2602)	0.5929 (0.2818)**	0.5140 (0.2498)**
* D(*1) × *H*(0)	−0.5860 (0.1593)***	−0.6134 (0.1528)***	0.0027 (0.1860)	−0.3186 (0.1607)**	0.2759 (0.1941)	0.2759 (0.1941)	0.4456 (0.1886)**	0.2862 (0.1674)*
* D*(1) × *H*(1)	−0.8279 (0.1385)***	−1.0237 (0.1551)***	−0.3340* (0.1727)	−0.6939 (0.1636)***	0.4804 (0.1650)***	0.4804 (0.1650)***	0.9439 (0.1687)***	0.8735 (0.1750)***
Control variables	Age, benign, L. income	Age, benign, L. income	Age, benign, L. income	Age, benign, L. income	Age, benign, L. income	Age, benign, L. income	Age, benign, L. income	Age, benign, L. income
Obs.	402	352	402	352	402	352	402	352
Clusters	378	326	378	326	378	326	378	326

Robust standard errors between brackets

Estimation output using nearest distance neighbor matching analysis without replacement and with random sorting of the data, and time fixed effects

Asterisk levels denote 1 % significance (***), 5 % significance (**) and 10 % significance (*)

**Table 9:** Impact of tumor detection and treatment on work and income (estimation output of the nearest neighbor matching model)

	Model: unable to work		Model: has no paid labor		Model: personal income		Model: household income	
	Women	Men	Women	Men	Women	Men	Women	Men
Reference *D*(0) × *H*(0)								
* D*(0) × *H*(1)	−0.1552 (0.1032)	−0.1944 (0.0900)**	0.0516 (0.1138)	−0.1029 (0.0560)*	−0.3542 (0.2063)*	0.0886 (0.1007)	−0.0942 (0.1666)	0.0846 (0.1053)
* D*(1) × *H*(0)	−0.0637 (0.0742)	−0.1275 (0.0643)**	−0.0070 (0.0602)	0.0305 (0.0466)	−0.0563 (0.0926)	−0.0531 (0.0700)	−0.0511 (0.0763)	−0.0802 (0.0733)
* D*(1) × *H*(1)	−0.2992 (0.0632)***	−0.3297* (0.0643)**	−0.0048 (0.0474)	0.0304 (0.0439)	−0.0785 (0.0846)	−0.0634 (0.0658)	0.0105 (0.0687)	−0.1058 (0.0673)
Control variables	Age, age sq., benign, d65+	Age, age sq., benign, d65+	Age, age sq., benign, d65+	Age, age sq., benign, d65+	Age, age sq., benign, d65+	Age, age sq., benign, d65+	Age, age sq., benign, d65+	Age, age sq., benign, d65+
Obs.	427	380	402	352	360	341	387	343
Clusters	378	326	378	326	378	326	378	326

Robust standard errors between brackets

Estimation output using nearest distance neighbor matching analysis without replacement and with random sorting of the data, and time fixed effects

Asterisk levels denote 1 % significance (***), 5 % significance (**) and 10 % significance (*)

**Fig. 1:**
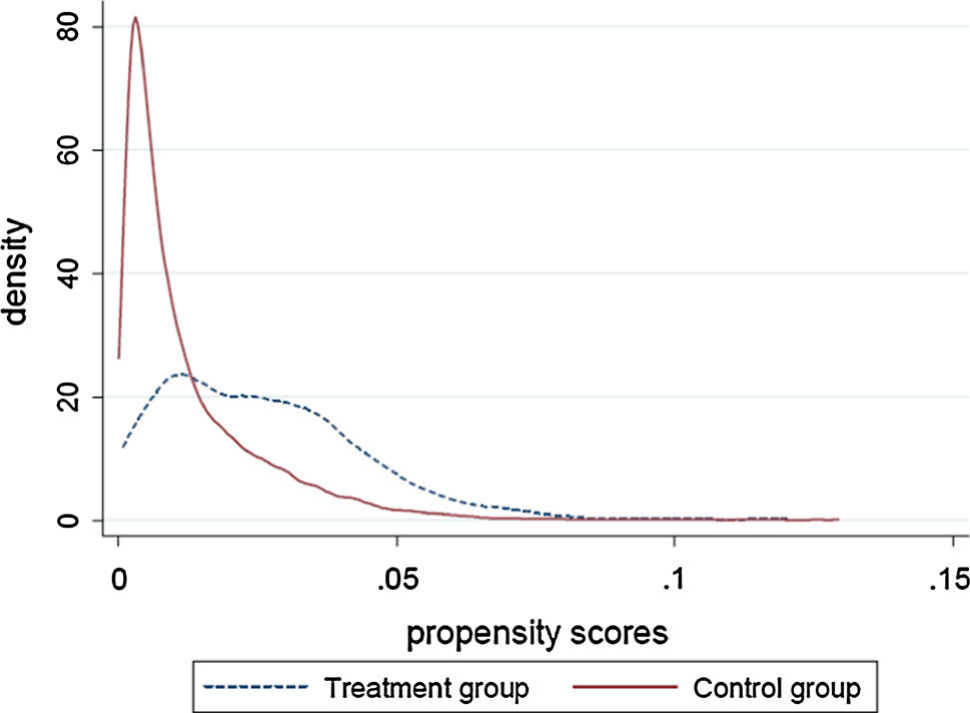
Check for the assumption of the common support by using matching analyses

In general, the estimates of the matching models with respect to psychosocial well-being are larger in magnitude in Table 8 than in Table 6. It seems that matching analyses overestimate the ‘trueʼ effects of tumor diagnosis. The overall picture of the matching models with respect to work and income is somewhat blurred. The estimates of Table 9, compared to those of Table 7, are underestimated for women, but overestimated for men. These findings could merely reflect the lack of comparability between the control group and the treatment group. Therefore, we check the assumption of common support (see Fig. 1). The assumption implies strong overlap in the covariate distribution of the case and control group, so that for each case individual a comparable control individual can be allocated [25, 26]. Having a rich set of individual and life-style determinants (Tables 10 and 11 in Appendix), Fig. 1 plots the overlap in covariate distributions by probability of treatment assignment. We observe that these covariate distributions do not overlap properly owing to the small number of control individuals available for high propensity scores. Consequently, case individuals rely on a small set of control individuals at the middle and upper tail of the propensity score values. In line with the work of Black and Smith [5], we argue that the support condition holds only weakly. A potential solution offered by Black and Smith ([5], p. 110 and p. 118) is then to only estimate the impacts of tumor detection for the “thick support” region in order to reduce selection (into the treatment) bias. In this region we observe a substantial number of observations in both the case and control group. Using the specification of Black and Smith [5], in our case, the thick support region is defined as those propensity score values below $\hat{P}(X) < 0.20$. Indeed, the majority of case individuals with relatively high propensity scores are then dropped from the analysis. Other matching estimators that weigh the control individuals in the matching process (e.g. the Epanechnikov kernel-matching estimator) do not significantly alter the estimates nor do they offer a solution to fit common support. We argue in line with Black and Smith [5] that it is not an option to allocate higher weights to control individuals who are less similar to case individuals (i.e. above $\hat{P}\left( X \right) > 0.20$) in order to construct appropriate counterfactual outcomes. These results, including balancing tests, are available from the authors upon request. We conclude that our findings do not support matching analysis.

**Table 10:** Information on individual characteristics (full sample, *N* = 850)

Variable	Obs	Mean	SD	Min	Max
Gender (male = 1)	850	0.4871	0.5001	0	1
Age	850	60.3	14.5	16	86
Civil status					
Married	850	0.6259	0.4842	0	1
Separated	850	0.0106	0.1024	0	1
Divorced	850	0.1318	0.3384	0	1
Widow or widower	850	0.0894	0.2855	0	1
Never been married	850	0.1424	0.3496	0	1
Household members	850	0.4224	0.8839	0	5
Living-at-home children	850	21.576	10.781	1	7
Type of dwelling					
Self-owned dwelling	850	0.6576	0.4748	0	1
Rental dwelling	850	0.3341	0.4720	0	1
Cost-free dwelling	850	0.0082	0.0904	0	1
Unknown (missing)	850	0.0000	0.0000	0	0
Degree of urbanization					
Extremely	849	0.1249	0.3307	0	1
Very	849	0.2603	0.4391	0	1
Moderately	849	0.2615	0.4397	0	1
Slightly	849	0.2285	0.4201	0	1
Not	849	0.1249	0.3307	0	1
Highest level of education with diploma					
Primary school	848	0.1297	0.3362	0	1
Pre-vocational secondary education	848	0.3196	0.4666	0	1
Higher secondary and pre-university education	848	0.0825	0.2754	0	1
Vocational education	848	0.2017	0.4015	0	1
Higher vocational education	848	0.1981	0.3988		
University	848	0.0684	0.2526	0	1
Occupational status					
Paid employment	850	0.2800	0.4493	0	1
Work or assist family business	850	0.0059	0.0765	0	1
Self-employed	850	0.0482	0.2144	0	1
Job seeker following job loss	850	0.0176	0.1317	0	1
First-time jobseeker	850	0.0000	0.0000	0	0
Exempted from job seeking following job loss	850	0.0059	0.0765	0	1
Attends school or university	850	0.0176	0.1317	0	1
Takes care of the housekeeping	850	0.0871	0.2821	0	1
Pensioner	850	0.4247	0.4946	0	1
Has (partial) work disability	850	0.0635	0.2441	0	1
Receives unemployment benefit	850	0.0059	0.0765	0	1
Performs voluntary work	850	0.0318	0.1755	0	1
Does something else	850	0.0118	0.1079	0	1
Is too young to have an occupation	850	0.0000	0.0000	0	0
Information on health insurance					
Individually or collectively?	761	0.3995	0.4901	0	1
Complementary health insurance?	761	0.8607	0.3465	0	1
How much is your own voluntary risk?					
I have no voluntary own risk	760	0.6592	0.4743	0	1
100 €	760	0.0816	0.2739	0	1
200 €	760	0.0987	0.2984	0	1
300 €	760	0.0145	0.1195	0	1
400 €	760	0.0026	0.0513	0	1
500 €	760	0.0197	0.1392	0	1
Don’t know	760	0.1237	0.3294	0	1

**Table 11:** Information on life-style (full sample, *N* = 850)

Variable	Obs	Mean	SD	Min	Max
Did you ever smoke? (no = 1)	764	0.6990	0.459	0	1
Do you still smoke?	850	0.1741	0.3794	0	1
Body mass index (BMI)	764	26.034	45.346	0	59
Drinking^a^					
Almost everyday	764	0.2749	0.4467	0	1
5 or 6 days per week	764	0.0641	0.2452	0	1
3 of 4 days per week	764	0.1034	0.3047	0	1
Once or twice a week	764	0.2094	0.4072	0	1
Once or twice a month	764	0.1008	0.3012	0	1
Once every 2 months	764	0.0615	0.2404	0	1
Once or twice a year	764	0.0877	0.283	0	1
Not at all over the last 12 months	764	0.0982	0.2977	0	1
Walking^b^	764	42.997	26.282	0	7
Fruit					
Never	764	0.0209	0.1433	0	1
1–3 times per month	764	0.0654	0.2475	0	1
1 time per week	764	0.0825	0.2752	0	1
2–4 times per week	764	0.1728	0.3783	0	1
5–6 times per week	764	0.1649	0.3714	0	1
Everyday	764	0.4935	0.5003	0	1
Whole-wheat					
Never	764	0.0131	0.1137	0	1
1–3 times per month	764	0.0118	0.108	0	1
1 time per week	764	0.0236	0.1518	0	1
2–4 times per week	764	0.0785	0.2692	0	1
5–6 times per week	764	0.1309	0.3375	0	1
Everyday	764	0.7421	0.4377	0	1
Fish					
Never	764	0.0969	0.296	0	1
1–3 times per month	764	0.2840	0.4512	0	1
1 time per week	764	0.3887	0.4878	0	1
2–4 times per week	764	0.2003	0.4005	0	1
5–6 times per week	764	0.0144	0.1192	0	1
Everyday	764	0.0157	0.1244	0	1
Meat					
Never	764	0.0209	0.1433	0	1
1–3 times per month	764	0.0366	0.188	0	1
1 time per week	764	0.0550	0.2281	0	1
2–4 times per week	764	0.2474	0.4318	0	1
5–6 times per week	764	0.3050	0.4607	0	1
Everyday	764	0.3351	0.4723	0	1
Vegetables (raw or cooked)					
Never	764	0.0236	0.1518	0	1
1–3 times per month	764	0.0458	0.2092	0	1
1 time per week	764	0.0641	0.2452	0	1
2–4 times per week	764	0.2474	0.4318	0	1
5–6 times per week	764	0.2644	0.4413	0	1
Everyday	764	0.3547	0.4787	0	1

^a^How often did you have a drink containing alcohol over the past 12 months?

^b^Over the last 7 days, on how many days did you spend time walking at least 10 min?

### Can anticipation bias the results?

It often takes several years before cancer is (or can be) detected. As such, individuals can already anticipate their diagnosis in the years before detection by, for example, changing their health insurance coverage or adapting their life-style. It is expected that the ‘trueʼ impact of tumor diagnosis is then underestimated. We test for anticipation by looking at short-run changes in health insurance coverage and life-style.

#### Health insurance coverage

In the Netherlands, health insurance is provided by privately owned health insurance companies, and basic coverage is compulsory for every individual. Besides this compulsory coverage, individuals may choose supplementary insurances. These insurances cover services that are deemed not essential medical care such as physiotherapy, dental care for adults or transport to the hospital. Individuals can only change their level of coverage once a year on January 1. Thus, it is impossible to change the premium rate or add supplementary insurances during the year. As such, in case of tumor detection and treatment in year t, individuals depend on the coverage they attained per January 1 of year *t*. If anticipation is likely, individuals should change their health insurance in year t compared to year *t* − 1. However, we do not find any differences between year *t* and year *t* − 1 with respect to changes in health insurance coverage (mean difference −0.0084; *T* value = −0.23); or changes from having no supplementary deductible to having one on top of the compulsory deductible (mean difference −0.0401; *T* value = −1.16).

#### Life-style

Life-style changes can also indicate anticipation (in year *t* − 1) or adaptation to the new situation (in year *t*). Using the rich set of life-style variables (information available to the authors upon request), we can easily check for significant changes in life-style between the control group and the treatment group. Again, we compute the mean difference between the case–control individuals. Most surprisingly, we do not find any short-run impact of tumor detection on changes in life-style. This includes no changes in: smoking (mean difference 0.0188; *T* value = 0.72); BMI (mean difference 0.1679; *T* value = 0.51); eating vegetables (mean difference −0.0001; *T* value = −0.01); eating fruit (mean difference −0.0154; *T* value = 1.48); eating whole-wheat (mean difference −0.0083; *T* value = −1.00); eating fish (mean difference 0.0044; *T* value = 0.21); or eating meat (mean difference 0.0058; *T* value = 0.56).

### Can other life changing events bias the results?

It is possible that other life changing events capture the effects estimated in the interaction effects. However, this is highly unlikely. First, these other events should cause a serious shock, and for more than one individual at the same time (i.e. it should drive the estimated average). This would indicate that tumor detection and treatment are not driving the sign and significance of the estimates, whereas other life-changing events are highly correlated with tumor diagnosis. To the best of our knowledge, we cannot recall such an event from the literature. And second, these other events cannot be a trend that covariates, for example, with age, as trends are captured in time fixed effects modeling, and age is controlled for.

### Can measurement error explain the results?

The literature indicates several possible types of measurement error. First, questionnaires are liable to recall bias, defined as the inability for respondents to correctly recall a past event at the time of the questionnaire. Bradley et al. [6] argue that (malignant) tumor diagnosis is a serious event that individuals do not forget. Second, questionnaires are also subject to issues with respect to misreporting. For example, an individual may tend to overstate or understate his/her ‘trueʼ feelings and emotions (see also [4, 8]. We deal with these issues of misreporting in the panel data model by controlling for reporting behavior intrinsic to the individual. To conclude, bias owing to misreporting issues on income is not an issue in LISS, as the panel is constructed by using population registries.

## Conclusion

The identification of causal effects in cancer research is often hampered by data constraints. This paper explored the beneficial features of combining national representative health surveys with administrative data on income and work. Owing to the data, we could treat tumor detection as a random event, a discontinuity, in order to compare a case–control group before and after tumor detection and treatment by using fixed effects models.

The results indicate that the negative effects of tumor detection and treatment on self-assessed health, happiness, and anxiety are highest for men. We also observe significant effects on self-reported health for women, but not on happiness or anxiety. Preference drift [13], can partially explain these results, as women who have been diagnosed with a benign tumor are happier in the year of tumor detection than the year before. It may also be that women, irrespective of their income position, adapt their feelings better to the new situation than men (i.e. women have better coping strategies), or that their emotions are driven by the same underlying mechanism of tumor formation (e.g. hormones). Previous literature on coping strategies explicitly accounting for gender differences is scarce. However, for women with a history of breast cancer, other studies found that the elderly like to hold on to their autonomy [20], that young women show purpose-seeking behavior, especially in their work [22], and that religion plays an important role in coping [2]. The latter theory on tumor formation is a medical question that we did not explore in this paper. Both theories are subject to further research.

Tumor detection and treatment affects work. The largest effects are found when hospitalization follows tumor diagnosis. In contrast to men, and in line with Bradley et al. [6], women drop out of the labor force more frequently in response to tumor detection. However, this does not affect their personal or household income. This short-run financial security is most likely driven by the Dutch compulsory health insurance coverage. Nonetheless, in the long-run employment decisions can also be affected by the way chronic health conditions are covered. Individuals may receive a 10 % premium discount in collective health insurance arrangements. Changing employers or dropping out of the labor force can lower or increase this premium rate. Carlsen et al. [9] already indicated an increased risk of quitting the labor force and taking early retirement pension or social security benefits up to 8 years after the initial diagnosis is made. Finally, Moran et al. [21] put our positive short-run outcomes in perspective by arguing that the long-run outcomes are worse for men who relapse. This is an important topic for further research.

Chronic diseases such as cancer may not only threaten survivors’ well-being and labor market participation; it may also thoroughly challenge the public health care system as well as individuals’ out-of-pocket expenses. Yabroff et al. [32, 33] estimated the costs associated with cancer care in the US. The authors indicate highest total care costs of about €3.18 billion in the first year of treatment for elderly patients (aged 65 and older) diagnosed with colorectal cancer, while the average 5-year net costs ranged from $20,000 for breast cancer care to more than $40,000 for brain (nervous system) cancer care. Like most studies on the costs of cancer care in the US, the authors did not capture the costs due to non-labor market participation owing to physical and/or psychosocial dysfunction for older and for younger cancer patients still in the labor force. They also did not include those cancer patients who are not eligible for health insurance, but, instead, they only focused on beneficiaries of the Medicare program. From our results we argue that health insurance coverage drives individuals’ financial well-being and these public costs and out-of-pocket expenses should not be neglected in further research.

To conclude, we find evidence against applying matching estimation techniques, but in favor of panel data models. First, matching analysis violates the assumption on common support, while tumor detection can be treated as a random event [6]. Second, comparing the aforementioned panel data estimates with those of the matching analyses, we find that matching overall overestimates the ‘true’ effects of tumor detection and treatment on subjective well-being. The effects of matching models are generally larger in magnitude. With respect to the estimates of the models on financial well-being, the picture is somewhat blurred. Compared with the panel data results, the estimates of the matching models are underestimated for women, but overestimated for men.

Notwithstanding the huge advantages of using panel data, there are also limitations to be considered. First, in our data, we cannot distinguish between recurrent and first ever cancer cases. We do not see this as a cause for concern for the relatively younger cases in our sample, as the official Dutch cancer registry indicates very low (negligible) incidence rates among this age group. Therefore, cancer incidence among young individuals is likely to be first ever cases. For the older age group, we acknowledge potential heterogeneous effects of recurrence of the tumor compared with first ever cases, in particular on psychosocial well-being. However, we also argue that the set of individuals with recurrent cases in our sample is low, which is supported by the high comparability between LISS panel and the official Dutch statistics on cancer incidence. Anyway, work and income should not be affected by this among the older age group, as also indicated by the insignificant results.

Second, we have no information on the staging or type of the cancer when it gets detected. Further policy debate could explore how nationally representative longitudinal questionnaires on well-being, work, and income can be enriched with health-related hospital data (i.e. the creation of a dataset which is “the best of both worlds”).

## Appendix

See Tables 10 and 11.

## Footnotes

1 Hereby, EU-12 Member States did not reach the initial 1985 target of 15 %.

2 Nonetheless, in recent debate there exists some controversy on the effectiveness of breast cancer screening in reducing mortality rates (e.g. KCE [18]; Canadian Task Force on Preventive Health Care [10].

3 We take into account that individuals can already have serious health impairments (i.e. other impairments than a tumor) needing hospitalization in the year before tumor diagnosis. In fact, we distinguish between $D\left( 0 \right) \times H(1)$ and $D\left( 0 \right) \times H\left( 0 \right)$.

4 Note that we do not have information before the year 2007, or after the year 2012. As such, the data is left and right censored.
